# Successful treatment of a patient with recurrent infection of *Chromobacterium violaceum*

**DOI:** 10.1186/s12879-021-06216-2

**Published:** 2021-05-26

**Authors:** Lijuan Lang, Menglei Wang, Xiaowen Huang, Hao Zhou, Zaigao Zhou, Liang Huang, Huanxin Zheng, Kang Zeng, Li Li

**Affiliations:** 1 Department of Dermatology, Southern Medical University Nanfang Hospital, Guangzhou, 510515 China; 2 Department of Infection Management, Southern Medical University Nanfang Hospital, Guangzhou, 510515 China

**Keywords:** Chromobacterium violaceum, Infection, Next-generation sequencing, Antimicrobial treatment

## Abstract

**Background:**

*Chromobacterium violaceum* (*C. violaceum*) is a Gram-negative saprophytic bacterium that is widespread in tropical and subtropical environments, and belongs to conditional pathogenic bacteria. Human infection with *C. violaceum* is rare, and this can be fatal when the diagnosis and treatment are delayed, especially recurrent infection patients. Since clinicians lack the knowledge for *C. violaceum,* rapid diagnosis and early appropriate antimicrobial treatment remains challenging.

**Case presentation:**

A 15-year-old male student was hospitalized for dark abscess, pustules, severe pain in both legs, and fever for 11 days. There were pustules with gray-white pus and red infiltrating plaques on the back, and the subcutaneous nodules could be touched in front of both tibias, with scab, rupture and necrotic tissue of the lower limb. The patient’s condition rapidly progressed. Therefore, next-generation sequencing (NGS), pustular secretion and blood culture were concurrently performed. The final diagnosis for this patient was *C. violaceum* infection by NGS. However, no bacterial or fungal growth was observed in the pustular secretion and blood culture. After 4 weeks of treatment, the patient was discharged from the hospital without any complications associated with *C. violaceum* infection.

**Conclusion:**

Rapid diagnosis and early appropriate antimicrobial treatment is the key to the successful treatment of *C. violaceum* infection, especially in patients with sepsis symptoms. This case highlights that NGS is a promising tool for the rapid diagnosis of *C. violaceum* infection, preventing the delayed diagnosis and misdiagnosis of *C. violaceum* infection in patients who tested negative for pustular secretion and blood culture.

## Background

*Chromobacterium violaceum* (*C. violaceum*) is a Gram-negative facultative anaerobic bacteria, which is slender, motile, sporeless and rod-shaped [1]. Furthermore, its colony is round, medium-sized and uniformly smooth, and it produces a chemical antioxidant compound called violacein, which makes the colony dark purple or even black [2]. In addition, it naturally resides in soil and stagnant water in tropical and subtropical regions [3]. *C. violaceum* was first identified in 1881. In 1905, Woolley isolated *C. violaceum* from a diseased buffalo in the Philippines, and described its pathogenicity for the first time [4]. In 1927, Malaysia first reported human cases of infection [4, 5]. More than 200 cases of infection with *C. violaceum* have been reported, and most of which are in Southeast Asia and the eastern United States [2, 6]. Pathogens enter through fractured or worn skin, causing local skin damage, urinary tract infection, pneumonia, severe sepsis with metastatic abscess, and septic shock, which rapidly develops to death due to multiple organ failure [1, 7, 8]. The next-generation sequencing (NGS) test uses high-throughput sequencing technology based on the BGISEQ-50/500 sequencing platform to analyze the microbial nucleic acid sequences in the samples, and identify suspicious pathogenic microorganisms by comparing with the nucleic acid sequences of existing microorganisms in the database. The detection range included 3446 bacteria (including 104 mycobacteria and 45 mycoplasma/chlamydia), 1515 DNA viruses, 206 fungi and 140 parasites with a known genome sequence. The detection process included the following: nucleic acid extraction, library construction, sequencing, information analysis, report interpretation, etc. We report a new case of bacteremia caused by *C. violaceum*. To the best of our knowledge, this is the first case, in which the patient successfully survived after being infected with *C. violaceum* for two times, and the first case, in which *C. violaceum* was diagnosed by NGS.

## Case presentation

**Fig. 1:**
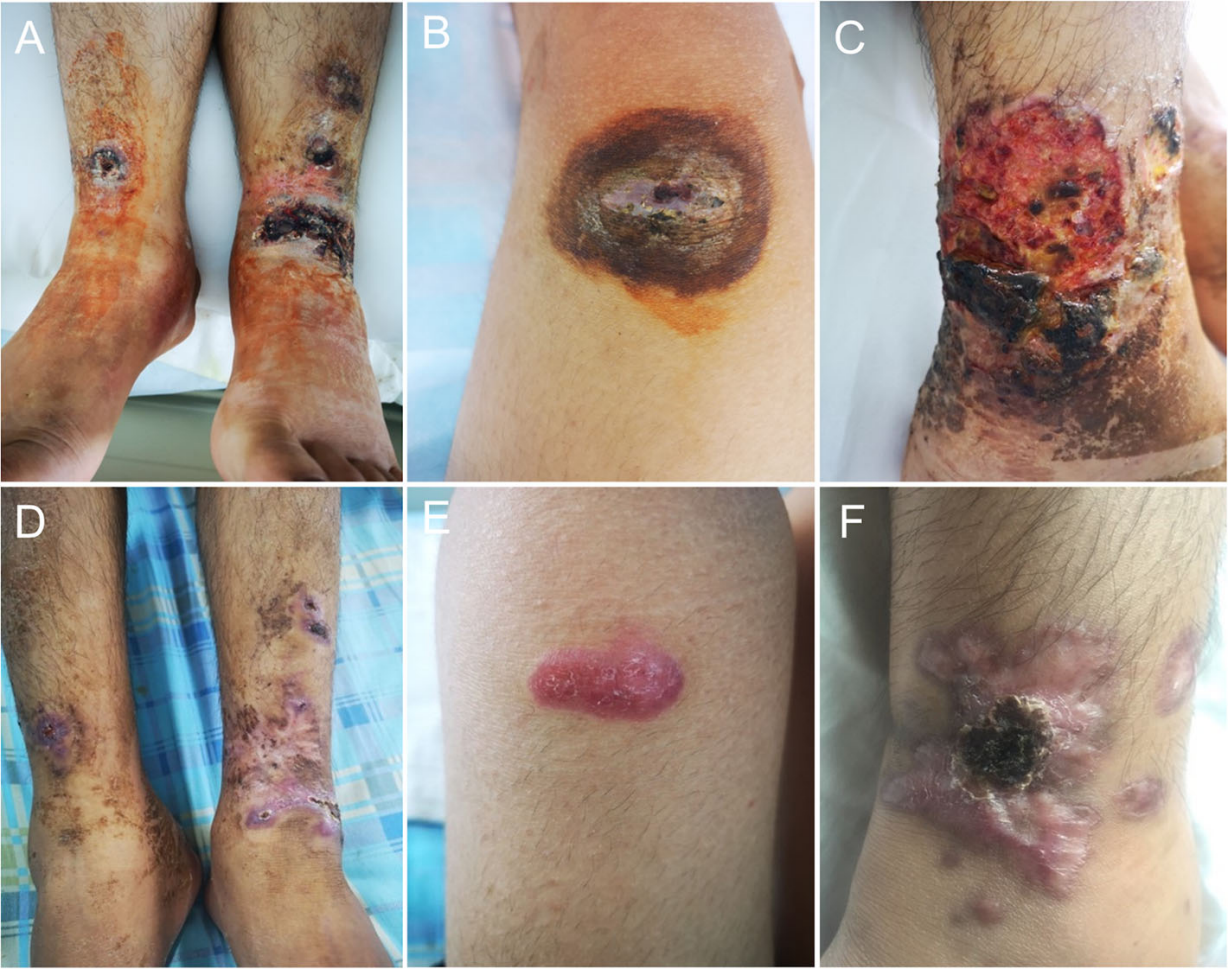
**A**, **B** and **C** shows multiple ulcers of the lower limbs with erosion, scab and necrosis. **D**, **E** and **F** shows the ulcers that have healed, and the scar formation after treatment

A 15-year-old male student was hospitalized for dark abscess, pustules, severe pain in both legs, and fever for 11 days (Fig. 1A, B and C). The patient developed pustules on both legs after scratching while working in the field, and this was accompanied by chills and fever, with a maximum temperature of 40 °C. Then, the pustules spread to the abdomen, back and upper arms. The lower legs were markedly swollen, and the pustules at the ankles were broken and ulcerated, forming purple and black scabs. This patient was unable to walk and sit in a wheelchair due to pain.

### Physical examination

There were pustules with gray-white pus and red infiltrating plaques on the back, and the subcutaneous nodules could be touched in front of both tibias, with scab, rupture and necrotic tissues of the lower limb. The liver and spleen did not touch. The patient’s consciousness, cranial nerve motor system, sensory system reflex, meningeal stimulation sign, and autonomic nerve function were examined. No positive signs were found, and organic lesions of the nervous system and septic shock were excluded.

**Fig. 2:**
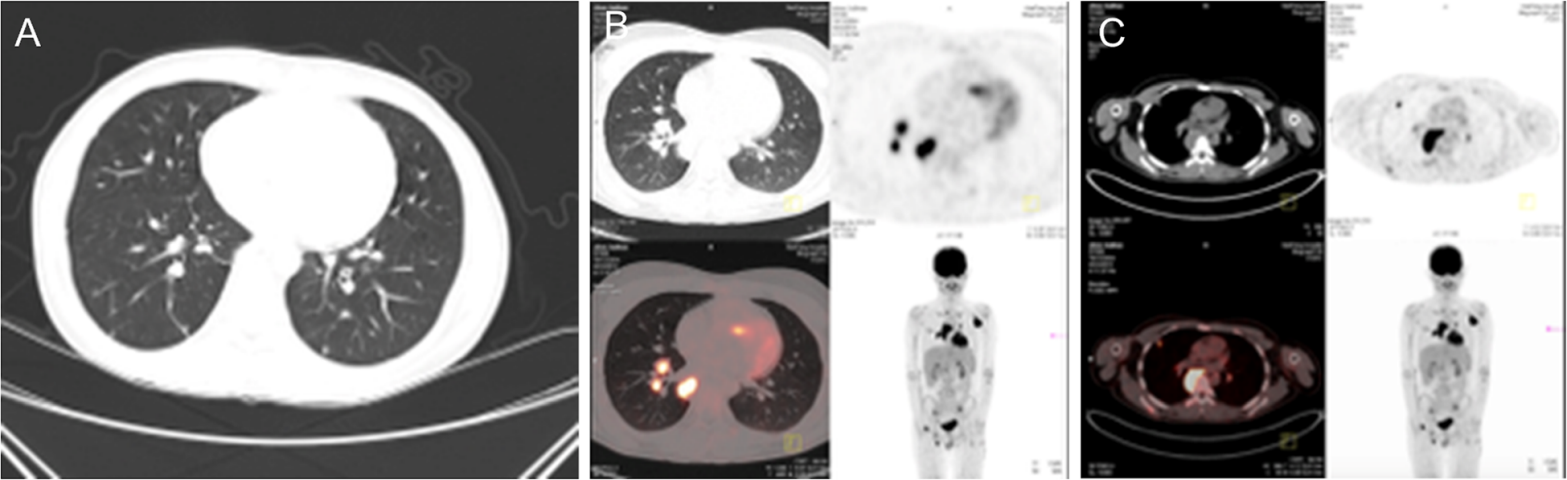
**A** The computed tomography scan of the chest shows the posterior mediastinal occupation. **B** and **C** The positron emission tomography-computed tomography scan reveals the hypermetabolic lesions in multiple parts of the body

**Fig. 3:**
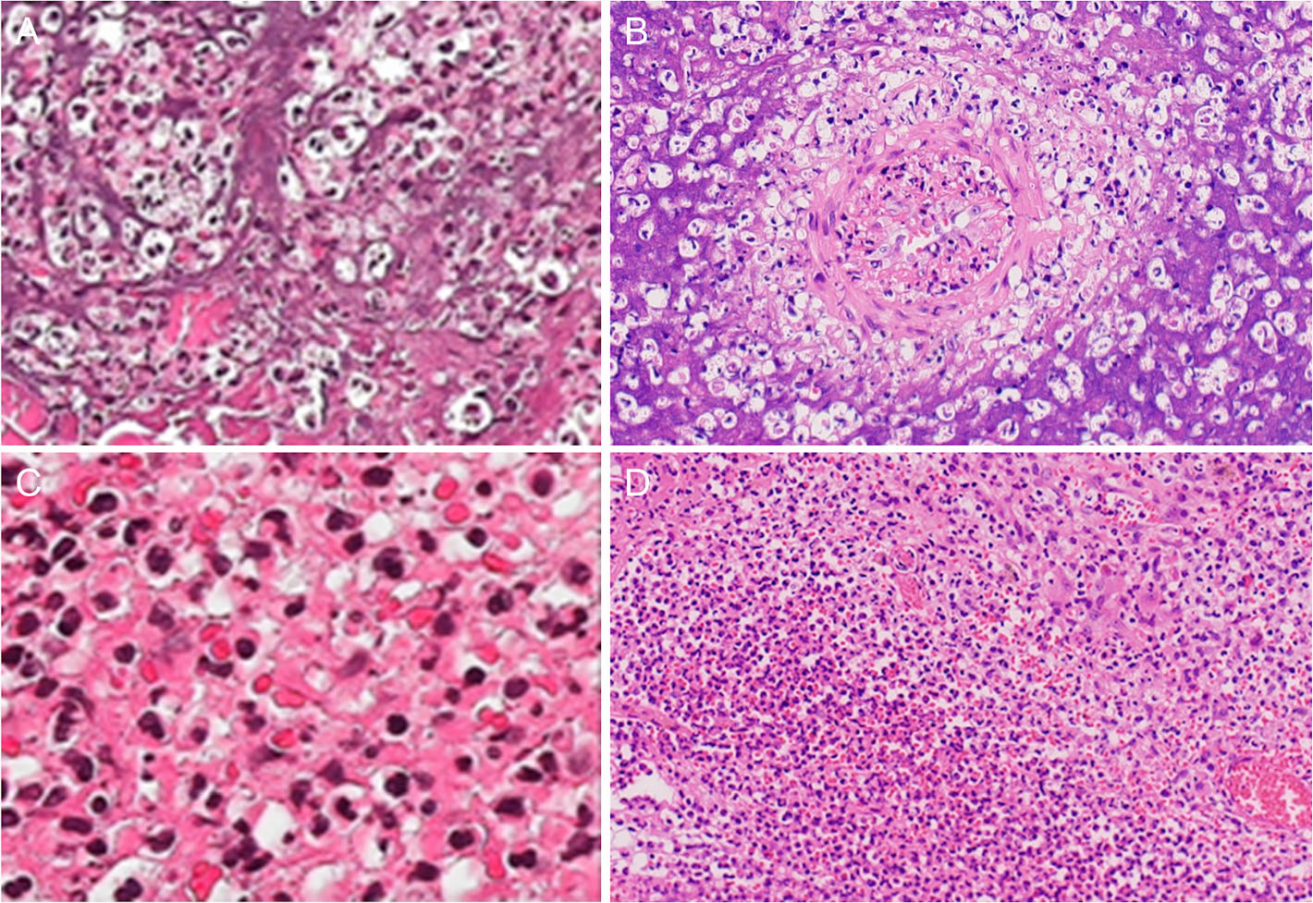
The pathological images of the back, **A** (× 400) and **B** (× 100), and abdomen, **C** (× 400) and **D** (× 100), showing the inflammation with abscess formation

After admission, the following results were recorded: hemoglobin, 113 g/L; white blood cell (WBC) count, 20.83 × 10^9^/L; lymphocyte (LYM) count, 0.52 × 10^9^/L; NEU, 19.75 × 10^9^/L; platelet (PLT), 112 × 10^9^/L; albumin (Alb), 30.00 g/L; erythrocyte sedimentation rate (ESR), 30 mm/hour; C-reactive protein (CRP), 247.81 mg/L. The enhanced computed tomography (CT) of the chest revealed a slightly low-density shadow on the bilateral frontal and parietal subcutaneous, considering the possibility of abscess, and on the posterior mediastinal occupation, considering tumorous lesions, while lymphoma was not excluded (Fig. 2A). Furthermore, the autoantibodies, vasculitis binomial, human immunodeficiency virus (HIV) detection, purified protein derivative (PPD) test, T-SPOT, hypertrophy reaction, and fungal D-glucan were negative. The surgical biopsy of the back and abdomen revealed inflammation with abscess formation (Fig. 3). However, no bacterial or fungal growth was observed in the pustular secretion and blood culture. The NGS results for the tissue revealed the following detection sequence (Table 1), indicating that the patient was infected with *C. violaceum*. Hence, the investigators immediately switched to the experimental treatment of 0.4 g of amikacin VD, 12 per hour, and 1 g of meropenem IV, six per hour, supplemented with nutritional support. The patient provided the blood culture and drug sensitivity results at the time of hospitalization in 2016, which revealed that the lower limb pus culture was *C. violaceum* infection. The patient was contacted for more details on the 2016 hospitalization data, but the patient lost this information due to the long period of time. The antimicrobial tests revealed that this was sensitive to amikacin, gentamicin, meropenem, norfloxacin, cefepime, imipenem, minocycline, compound neonamine and levofloxacin, was intermediate-resistant to cefoperazone/sulbactam and aztreonam, and resistant to ceftazidime and piperacillin/tazobactam. The antibiotics chosen by the investigators was consistent with the drug sensitivity results. After 4 weeks of treatment, the patient’s temperature dropped to the normal range. The chest enhanced CT re-examination revealed that the bilateral inflammatory nodules were absorbed, when compared pre-treatment. The re-examination for blood routine, erythrocyte sedimentation rate (ESR), C-reactive protein (CRP) and other indicators basically returned to normal. Furthermore, the whole body positron emission tomography-computed tomography (PET-CT) examination revealed that there were multiple enlarged lymph nodes in the left axilla, bilateral hilum and mediastinum (subcarinal lymph nodes). Moreover, the metabolism significantly increased, and some lesions in the left axilla were accompanied by calcification. It was suggested that lymphoma should be excluded by the biopsy of hypermetabolic lesions. Multiple nodular hypermetabolic lesions were observed in multiple parts of the patient’s bones, and changes in bone density were considered to be correlated to infection. Multiple joints in the body have increased the metabolism, considering the multiple arthritis. In addition, hypermetabolism was observed in other parts of the body, and inflammatory lesions were considered (Fig. 2B and C). The patient’s skin rash improved (Fig. 1D, E and F), and the patient went to an oncology hospital for further treatment.

**Table 1:** The next-generation sequencing report

Type	Category	Detection sequence	Species	Detection sequence
G^−^	Chromobacterium	562	Chromobacterium violaceum	456
G^+^	Lactobacillus	26	Lactobacillus crispatus	24
G^−^	Acinetobacter	3	–	–
G^+^	Staphylococcus	3	–	–
Fungus	Malassezia	8	Malassezia japonica	7
Viruses	–	–	–	–
Parasite	–	–	–	–
M.tuberculosis	–	–	–	–
UU/CT	–	–	–	–

Notes: Detection sequence: The number of strictly aligned sequences of the microorganism detected at the genus/species level; −: Indicates that the information is not available; G^+^: Gram-positive bacteria; G^−^: Gram-negative bacteria

## Discussion

Although human infections caused by *C. violaceum* are rare, more than 200 cases have been reported worldwide [2]. The clinical manifestations of *C. violaceum* infection greatly vary, which ranges from mild fever, diarrhea, liver abscess, lung abscess, urinary tract infection and abdominal abscess, to rapid fatal sepsis [9]. The relevant clinical manifestations depend on the involved organs. The patient presented with high fever and occasional chills, severe pain in the whole body, and obvious tenderness in various parts of the body and joints, and was unable to walk. Patients with recurrent infection of *C. violaceum* that involve multiple parts and organs of the body are rare, especially cases that involve multiple bones and joints, which may be correlated to severe pain and the inability to walk.

*C. violaceum* is generally considered to be non-pathogenic [10], and immunodeficiency may be the potential cause of infection. Patients with chronic granulomatous disease and neutrophil dysfunction are generally susceptible to *C. violaceum* infection [11]. The present patient was infected with this bacterium in 2016. After being hospitalized for nearly a month, the patient became better, and was discharged. Then, the patient was re-diagnosed with *C. violaceum* infection after 3 years. However, it could not be confirmed whether this was a recurrence caused by incomplete treatment at the first infection or reinfection. It is possible that the first antibiotic treatment course was inadequate, leading to recurrence. However, the patient’s examination results took into account tumorous lesions, which may have some immune deficiencies that led to the reinfection.

Most cases are confirmed by bacteria cultured from body fluids and blood. To our knowledge, the present patient is the first confirmed case of *C. violaceum* infection by NGS, and the first case that successfully survived after infection with *C. violaceum* for two times. This is possibly due to the use of a large number of empirical antibiotics or other influences, and because *C. violaceum* could not be cultured in the patient’s secretion, blood, urine and feces for a number of times. NGS is increasingly being used in the pathogen diagnosis of difficult and critical infections. This provides a new and effective approach for the diagnosis of infections with rapid progression and high mortality, such as *C. violaceum*. Due to the rare case of infection with *C. violaceum*, the mechanism of pathological damage remains unclear, and clinicians lack the knowledge of *C. violaceum*. As an emerging and “underdiagnosed” pathogen, there are no clinical diagnostic guidelines for *C. violaceum*. According to the cases reported in the literature, it was found that the increase in WBC, NEU, ESR and CRP, and the decrease in ALB are of diagnostic significance. However, the diagnoses evidence of this bacterium mainly depends on clinical manifestations and detection of infected bacteria. We report this case with the hope of helping clinicians further understand the infection of *C. violaceum*, and providing clinical information to improve the diagnosis and treatment of the *C. violaceum* infection.

## Conclusions

The infections caused by *Chromobacterium violaceum* may be treated successfully, if prompt proper treatment is started. For this, the clinicians should be aware of rapid diagnosis will become crucial. Next-generation sequencing may offer higher sensitivity for pathogen detection, enabling earlier diagnosis and more targeted antimicrobial therapy.

## Data Availability

All data generated and analyzed during the study were included in the published article.

## Abbreviations

*C. violaceum*: Chromobacterium violaceum
NGS: Next-generation sequencing
WBC: White blood cells
LYM: Lymphocytes
PLT: Platelets
ALB: Albumin
ESR: Erythrocyte sedimentation rate
CRP: C-reactive protein
CT: Computed tomography
HIV: Human immunodeficiency virus
PPD: Purified protein derivative
PET-CT: Positron emission tomography-computed tomography
